# Supplementary material for: Content-rich biological network constructed by mining PubMed abstracts
Source: BMC Bioinformatics. 2004 Oct 8;5:147. doi: 10.1186/1471-2105-5-147 (PMC528731; doi:10.1186/1471-2105-5-147)
Supplement: Additional File 2 — The original results of the above study (non-essential files are deleted to keep the file size under the limit set by BMC bioinformatics). [file 1471-2105-5-147-S2.bz2 › chilibotAdditionalFile2/dip50/211ID7768894E717/html/CSNK2A1_CSNK2B.html]

 


 **CSNK2A1** and **CSNK2B** 
  
Found 143 abstracts in PubMed, retrieved 50.  
 

 What does Google say? 
 PDF only 
| .edu only 

---

**Interactive relationship** (e.g. stimulation, inhibition, etc)

**Neutral relationship**- Phosphorylation of EYFP 4E BP1 by the mitogen activated protein kinase ERK2, but not by casein kinase CK II  [ **CSNK2B** ]   [ **CSNK2A1** ] , also attenuated the interaction.  Ref: 11404183 Int J Biochem Cell Biol, 2001
- We found that the stress induced kinase casein kinase CK II  [ **CSNK2B** ]   [ **CSNK2A1** ]  phosphorylates the Ser276 immediately preceding the tyrosine motif, increasing AQP4 mu 3A interaction and enhancing AQP4 lysosomal targeting and degradation.  Ref: 11742978 EMBO J, 2001
- A polyclonal antibody was generated that binds to bacterially expressed p53 only when phosphorylated in vitro by CK II  [ **CSNK2B** ]   [ **CSNK2A1** ] .  Ref: 9501176 Proc Natl Acad Sci U S A, 1998
- CK II  [ **CSNK2B** ]   [ **CSNK2A1** ]  activity was assayed by incubating CK II  [ **CSNK2B** ]   [ **CSNK2A1** ]  with dephosphorylated casein and gamma 32P ATP.  Ref: 10072895 Zhongguo Yao Li Xue Bao, 1997
- CK II  [ **CSNK2B** ]   [ **CSNK2A1** ]  activity was assayed by incubating CK II  [ **CSNK2B** ]   [ **CSNK2A1** ]  with dephosphorylated casein and gamma 32 P ATP.  Ref: 9812723 Zhongguo Yao Li Xue Bao, 1996
- Second, directed disruption of a protein kinase casein kinase CK II  [ **CSNK2B** ]   [ **CSNK2A1** ]  site, located within the EKLF interaction domain, abolishes EKLF transactivation and in vivo competition activity.  Ref: 9722526 J Biol Chem, 1998
- The association of CK II  [ **CSNK2B** ]   [ **CSNK2A1** ]  with the cardiac SR, even after EGTA extraction at alkaline pH, is demonstrated using antibodies against CK II  [ **CSNK2B** ]   [ **CSNK2A1** ] .  Ref: 10398759 J Membr Biol, 1999
- The three casein kinase II CK II  [ **CSNK2B** ]   [ **CSNK2A1** ]  phosphate acceptors p35, p17 and p15 in the Superdex CK II  [ **CSNK2B** ]   [ **CSNK2A1** ]  fraction prepared from a 1.5 M NaCl extract of porcine liver were selectively purified by glycyrrhizin GL affinity column chromatography HPLC as a heterocomplex associated with CK II  [ **CSNK2B** ]   [ **CSNK2A1** ] .  Ref: 10706406 Biol Pharm Bull, 2000

**Non-interactive relationship** (e.g. studied together, co-existance, homology, etc.)

- ii natural anti oxidative compounds with polyphenols, but not GL and GA, act as potent CK II  [ **CSNK2B** ]   [ **CSNK2A1** ]  suppressors.  Ref: 10443459 Biol Pharm Bull, 1999
- Rhodnius CK II  [ **CSNK2B** ]   [ **CSNK2A1** ]  is strongly inhibited by heparin Ki = 9 nM and uses ATP Km = 36 microM or GTP Km = 86 microM as phosphate donors.  Ref: 12110292 Insect Biochem Mol Biol, 2002
- Despite many reports showing that CK I mediates phosphorylation of neurofilaments, CK II  [ **CSNK2B** ]   [ **CSNK2A1** ]  may phosphorylate NF L in vivo.  Ref: 10428477 FEBS Lett, 1999
- Because phosphorylation of p53 at the casein kinase CK II  [ **CSNK2B** ]   [ **CSNK2A1** ]  site activates p53 for DNA binding function in vitro, we sought to determine the in vivo relevance of phosphorylation at this site after UV and gamma radiation.  Ref: 9501176 Proc Natl Acad Sci U S A, 1998
- In contrast to previous studies, which locate CK II  [ **CSNK2B** ]   [ **CSNK2A1** ]  phosphorylation sites exclusively to the C terminal PEST sequence of Ikappa Balpha, we observed efficient phosphorylation of serine 32 in Ikappa Balpha by the purified endogenous CK II  [ **CSNK2B** ]   [ **CSNK2A1** ]  complex.  Ref: 9914500 Eur J Biochem, 1999
- An average of about two of the four potential sites were phosphorylated in MIIB beta F47, while in MIIB alpha F47 all five sites could be fully phosphorylated by CK II  [ **CSNK2B** ]   [ **CSNK2A1** ] .  Ref: 9485326 Biochemistry, 1998
- Furthermore, these three gbPLA2s were phosphorylated by casein kinase II CK II  [ **CSNK2B** ]   [ **CSNK2A1** ]  in vitro and GL inhibited the CK II  [ **CSNK2B** ]   [ **CSNK2A1** ]  mediated stimulation of their enzyme activities in vitro.  Ref: 9657040 Biol Pharm Bull, 1998
- The activated CK II  [ **CSNK2B** ]   [ **CSNK2A1** ]  phosphorylates several cellular and viral proteins in HIV 1 infected human MOLT 4 cells, and also phosphorylates HIV 1 structural proteins, including recombinant reverse transcriptase rRT.  Ref: 9654140 FEBS Lett, 1998
- CONCLUSION AG34, AG372, and AG1112 were potent inhibitors of CK II  [ **CSNK2B** ]   [ **CSNK2A1** ] , and the inhibitory action of AG34 was noncompetitive with casein and ATP.  Ref: 10072895 Zhongguo Yao Li Xue Bao, 1997
- Purified protein kinase A PKA, casein kinase II CK II  [ **CSNK2B** ]   [ **CSNK2A1** ] , and protein kinase C PKC phosphorylated the recombinant AGT protein with a stoichiometry of 0.1.28, and 0.44 mol phosphate incorporated mol protein, respectively.  Ref: 10667577 Cancer Res, 2000
- Since CK II  [ **CSNK2B** ]   [ **CSNK2A1** ]  is central in all these events, which are specifically affected by ethanol, the role of nuclear CK II  [ **CSNK2B** ]   [ **CSNK2A1** ]  is investigated in the present study.  Ref: 9178953 Neurochem Res, 1997
- 6.6 nM, which is about 30 fold lower than that observed with CK II  [ **CSNK2B** ]   [ **CSNK2A1** ]  beta.  Ref: 11226880 J Biochem Tokyo , 2001
- Three effective phosphate acceptors 3 5 and 13 kDa polypeptides for casein kinase II CK II  [ **CSNK2B** ]   [ **CSNK2A1** ]  in the Superdex CK II  [ **CSNK2B** ]   [ **CSNK2A1** ]  fraction prepared from a 0.5 M NaCl extract of bamboo shoots were selectively purified by glycyrrhizin GL affinity column chromatography HPLC.  Ref: 10443459 Biol Pharm Bull, 1999
- and ii the selective inhibition of CK II  [ **CSNK2B** ]   [ **CSNK2A1** ]  mediated activation of HIV 1 RT by potent CK II  [ **CSNK2B** ]   [ **CSNK2A1** ]  inhibitors may be involved in the mechanism of their anti HIV 1 effects at the cellular level.  Ref: 10549869 Biol Pharm Bull, 1999
- These phosphorylations are selectively inhibited by CK II  [ **CSNK2B** ]   [ **CSNK2A1** ]  inhibitors, such as quercetin, oGA a glycyrrhetinic acid derivative and NCS chrom an enediyne containing antibiotic.  Ref: 9654140 FEBS Lett, 1998
- this site is within casein kinase CK II  [ **CSNK2B** ]   [ **CSNK2A1** ]  consensus sequence, although showing some similarity to CKI sequence.  Ref: 10702244 J Biol Chem, 2000
- Phylogenetically conserved CK II  [ **CSNK2B** ]   [ **CSNK2A1** ]  phosphorylation site of the murine homeodomain protein Hoxb 6.  Ref: 10327653 J Exp Zool, 1999
- METHODS CK II  [ **CSNK2B** ]   [ **CSNK2A1** ]  was partially purified from rat livers by sequential DE52 and heparin Sepharose chromatography.  Ref: 10072895 Zhongguo Yao Li Xue Bao, 1997
- These data indicate that CK II  [ **CSNK2B** ]   [ **CSNK2A1** ]  is activated in a fertilization dependent process.  Ref: 10319435 Insect Biochem Mol Biol, 1999
- It is maximally phosphorylated at the third day of embryogenesis by CK II  [ **CSNK2B** ]   [ **CSNK2A1** ]  and then its phosphorylation decreases to a basal level by the time of first instar eclosion.  Ref: 10319435 Insect Biochem Mol Biol, 1999
- Phosphorylation of the MP was decreased by addition of kinase inhibitors such as heparin, suramin and quercetin, which are known to be effective for casein kinase II CK II  [ **CSNK2B** ]   [ **CSNK2A1** ] .  Ref: 10900049 J Gen Virol, 2000
- Immunological detection by western analysis using CK II  [ **CSNK2B** ]   [ **CSNK2A1** ]  antibodies showed no alteration in the quantity of enzyme.  Ref: 9178953 Neurochem Res, 1997
- These results, therefore, indicate that the purified kinase is a CK II  [ **CSNK2B** ]   [ **CSNK2A1** ]  protein kinase and may be involved in the regulation of DNA topoisomerase I activity.  Ref: 12243354 Mol Cells, 2002
- These results suggest that the anti HIV 1 effect of oGA may be involved in the selective inhibition of the CK II  [ **CSNK2B** ]   [ **CSNK2A1** ]  mediated stimulation of HIV 1 RT at the cellular level.  Ref: 9881639 Biol Pharm Bull, 1998
- CONCLUSION Our results suggest that CK II  [ **CSNK2B** ]   [ **CSNK2A1** ]  plays an important role in cellular proliferation and the mechanism of its activation.  Ref: 12017291 Anticancer Res< MedlineTA>Anticancer Res, 1998
- Two of them correspond to CK II  [ **CSNK2B** ]   [ **CSNK2A1** ]  and the other three have been called RAP 1, RAP II, and RAP III.  Ref: 10683252 Arch Biochem Biophys, 2000
- We studied a phosphate acceptor for casein kinase II CK II  [ **CSNK2B** ]   [ **CSNK2A1** ]  in chloroplasts, and found a 56 kDa protein p56 as an acceptor, which was partially purified from the stroma of spinach chloroplasts.  Ref: 9784844 Biochem Mol Biol Int, 1998
- The activity range of CK II  [ **CSNK2B** ]   [ **CSNK2A1** ]  was between 500 1500 U mg protein.  Ref: 12017291 Anticancer Res< MedlineTA>Anticancer Res, 1998
- METHODS CK II  [ **CSNK2B** ]   [ **CSNK2A1** ]  was partially purified from rat livers by sequential DE52 and heparin Sepharose chromatography.  Ref: 9812723 Zhongguo Yao Li Xue Bao, 1996
- Physical, biochemical and antigenic properties of this kinase identify it as casein kinase II CK II  [ **CSNK2B** ]   [ **CSNK2A1** ] .  Ref: 11861906 Nucleic Acids Res, 2002
- The endogenous kinase is identified as casein kinase II CK II  [ **CSNK2B** ]   [ **CSNK2A1** ]  based on its ability to use GTP as effectively as ATP, and its inhibition by La3.  Ref: 10398759 J Membr Biol, 1999
- Replacement of the CK II  [ **CSNK2B** ]   [ **CSNK2A1** ]  or PK C sites with Asp MIIB alpha F47 CK 5D or PK 4D strongly inhibited the filament assembly, with or without Mg 2, by significantly increasing the critical concentrations for assembly.  Ref: 10985790 Biochemistry, 2000
- An endogenous CK II  [ **CSNK2B** ]   [ **CSNK2A1** ]  can phosphorylate sarcalumenin, and in parallel to its phosphorylation the properties of the ryanodine receptor are modified.  Ref: 10398759 J Membr Biol, 1999
- Thus, RAP II and PK60 mainly phosphorylate P1beta and P2alpha whereas RAP I and CK II  [ **CSNK2B** ]   [ **CSNK2A1** ]  modify all of them.  Ref: 10683252 Arch Biochem Biophys, 2000
- Moreover, a derivative oGA of glycyrrhetinic acid GA and several polyphenol containing anti oxidative compounds quercetin, epigallocatechin gallate EGCG and two isoflavones, i.e.,, 7 trihydroxyisoflavone, 7 THI and 8 chloro, tetrahydroxyisoflavone 8C, THI inhibited the CK II  [ **CSNK2B** ]   [ **CSNK2A1** ]  mediated phosphorylation of 60S acidic ribosomal P proteins in vitro.  Ref: 10443459 Biol Pharm Bull, 1999
- AIM To study the action of tyrphostin on casein kinase CK II  [ **CSNK2B** ]   [ **CSNK2A1** ] .  Ref: 10072895 Zhongguo Yao Li Xue Bao, 1997
- ii the RDDP, DDDP and RNase H activity of rRT was stimulated about 2..1 and 3.9 fold, respectively, after full phosphorylation by CK II  [ **CSNK2B** ]   [ **CSNK2A1** ] .  Ref: 10549869 Biol Pharm Bull, 1999
- The same mutations also abolish phosphorylation of eIF5 when transfected into mammalian cells suggesting that CK II  [ **CSNK2B** ]   [ **CSNK2A1** ]  phosphorylates eIF5 at these two serine residues in vivo as well.  Ref: 11861906 Nucleic Acids Res, 2002
- and ii the selective inhibition of the CK II  [ **CSNK2B** ]   [ **CSNK2A1** ]  mediated stimulation of HIV 1 PR and RT by potent CK II  [ **CSNK2B** ]   [ **CSNK2A1** ]  inhibitors may be involved in their anti HIV 1 effects at the cellular level.  Ref: 10964683 Biochem Biophys Res Commun, 2000
- These results suggest that the MP of ToMV could be phosphorylated in plant cells by a host protein kinase that is closely related to CK II  [ **CSNK2B** ]   [ **CSNK2A1** ] .  Ref: 10900049 J Gen Virol, 2000
- In addition, the recombinant beta subunit of CF1 was phosphorylated when the subunit was incubated with CK II  [ **CSNK2B** ]   [ **CSNK2A1** ] .  Ref: 9784844 Biochem Mol Biol Int, 1998
- It was found that i the purified recombinant RT rRT functioned as an effective phosphate acceptor for CK II  [ **CSNK2B** ]   [ **CSNK2A1** ] .  Ref: 10549869 Biol Pharm Bull, 1999
- These results suggest that i CK II  [ **CSNK2B** ]   [ **CSNK2A1** ]  may be a host mediator responsible for stimulation of PR and RT in HIV 1 infected cells.  Ref: 10964683 Biochem Biophys Res Commun, 2000
- CK2A  [ **CSNK2A1** ] , CK2B  [ **CSNK2B** ] , Alcaligenes sp.  Ref: 10917884 J Microbiol Immunol Infect, 2000
- ii purified both proteins function as phosphate acceptors of CK II  [ **CSNK2B** ]   [ **CSNK2A1** ] .  Ref: 11041245 Biol Pharm Bull, 2000
- and iii oGA at one tenth the concentration of GL inhibits the CK II  [ **CSNK2B** ]   [ **CSNK2A1** ]  mediated stimulation of sPLA2 IIA activity.  Ref: 11558558 Biol Pharm Bull, 2001
- RAP II was further purified on phosphocellulose, heparin Sepharose, and P protein Sepharose, studied in detail, and compared with other acidic protein kinases, including RAP I, CK II  [ **CSNK2B** ]   [ **CSNK2A1** ] , and PK60.  Ref: 10683252 Arch Biochem Biophys, 2000
- For CK II  [ **CSNK2B** ]   [ **CSNK2A1** ] , five sites were identified within the COOH end of MIIB alpha F47 S PO4 L E L S PO4 D D D T PO4 E S K T S PO4 D V N E T Q P P Q S PO4 E.  Ref: 9485326 Biochemistry, 1998
- However, gamma radiation, which also stabilizes p53, did not result in phosphorylation at the CK II  [ **CSNK2B** ]   [ **CSNK2A1** ]  site.  Ref: 9501176 Proc Natl Acad Sci U S A, 1998
- Kinetic studies of AG34 on CK II  [ **CSNK2B** ]   [ **CSNK2A1** ]  showed that it was noncompetitive with casein and ATP.  Ref: 10072895 Zhongguo Yao Li Xue Bao, 1997
- The decrease in CK II  [ **CSNK2B** ]   [ **CSNK2A1** ]  activity against VT coincides with the beginning of VT proteolysis processing suggesting a possible relationship between protein phosphorylation and yolk degradation.  Ref: 10319435 Insect Biochem Mol Biol, 1999
- Furthermore, it was found that i purified sPLA2 IIA is phosphorylated by casein kinase II CK II  [ **CSNK2B** ]   [ **CSNK2A1** ]  in vitro.  Ref: 11558558 Biol Pharm Bull, 2001
- Both purified HMG1 2 functioned as phosphate acceptors for these two protein kinases CK I and PKC, but not phosphorylated by CK II  [ **CSNK2B** ]   [ **CSNK2A1** ] .  Ref: 11510483 Biol Pharm Bull, 2001
- These results suggest that i a GL affinity column is useful for effective purification of 60S acidic ribosomal P proteins from various mammalian cells as a heterocomplex associated with CK II  [ **CSNK2B** ]   [ **CSNK2A1** ] .  Ref: 10706406 Biol Pharm Bull, 2000
- Three polyphenol containing anti oxidant compounds catechin, epigallocatechin gallate EGCG and quercetin inhibited CK II  [ **CSNK2B** ]   [ **CSNK2A1** ]  activity phosphorylation of these ribosomal P proteins in a dose dependent manner in vitro.  Ref: 10706406 Biol Pharm Bull, 2000
- Based on the pattern of phosphorylation of the wildtype and two variant forms of eIF2B epsilon using casein kinase CK I, CK II  [ **CSNK2B** ]   [ **CSNK2A1** ] , or GSK 3 as well as that observed with skeletal muscle extracts, we conclude that the predominant eIF2B epsilon kinase in psoas muscle is GSK 3.  Ref: 10216953 Int J Biochem Cell Biol, 1999
- Characterization of secretory type IIA phospholipase A2 sPLA2 IIA as a glycyrrhizin GL binding protein and the GL induced inhibition of the CK II  [ **CSNK2B** ]   [ **CSNK2A1** ]  mediated stimulation of sPLA2 IIA activity in vitro.  Ref: 11558558 Biol Pharm Bull, 2001
- Casein kinase II CK II  [ **CSNK2B** ]   [ **CSNK2A1** ]  mediated stimulation of HIV 1 reverse transcriptase activity and characterization of selective inhibitors in vitro.  Ref: 10549869 Biol Pharm Bull, 1999
- RESULTS AG34 inhibited the activity of CK II  [ **CSNK2B** ]   [ **CSNK2A1** ]  with IC50 33 27 41 mumol.L 1.  Ref: 10072895 Zhongguo Yao Li Xue Bao, 1997
- In addition, as shown for animal and plant CK II  [ **CSNK2B** ]   [ **CSNK2A1** ] , gamma 32 P GTP was efficiently used as a phosphoryl donor.  Ref: 10900049 J Gen Virol, 2000
- We also identified consensus phosphorylation motifs for many cellular kinases, including PKA and CK II  [ **CSNK2B** ]   [ **CSNK2A1** ]  in the AGT protein.  Ref: 10667577 Cancer Res, 2000
- and iii CK II  [ **CSNK2B** ]   [ **CSNK2A1** ]  mediates the regulation of the physiological functions of 60S acidic ribosomal P proteins in growing plant cells.  Ref: 10443459 Biol Pharm Bull, 1999
- hondai on the activities of four distinct Ser Thr protein kinases two casein kinases CK I and CK II  [ **CSNK2B** ]   [ **CSNK2A1** ] , A kinase and C kinase were determined in vitro.  Ref: 12612451 Biol Pharm Bull, 2003
- The antibody showed that casein kinase I CK I as well as casein kinase II CK II  [ **CSNK2B** ]   [ **CSNK2A1** ]  phosphorylated Ser 473 in vitro, while neither GSK 3beta nor calcium calmodulin dependent protein kinase II did so.  Ref: 10428477 FEBS Lett, 1999
- However, no significant effect of the CK II  [ **CSNK2B** ]   [ **CSNK2A1** ]  mediated hLF phosphorylation on its DNA binding affinity was detected.  Ref: 11041245 Biol Pharm Bull, 2000
- The physiological significance of the casein kinase II CK II  [ **CSNK2B** ]   [ **CSNK2A1** ]  mediated phosphorylation of human immunodeficiency virus type 1 HIV 1 reverse transcriptase RT on its three enzymatic activities RNA dependent DNA polymerase RDDP, DNA dependent DNA polymerase DDDP and ribonuclease H RNase H was investigated in vitro.  Ref: 10549869 Biol Pharm Bull, 1999
- Biochemical characterization of recombinant HIV 1 reverse transcriptase rRT as a glycyrrhizin binding protein and the CK II  [ **CSNK2B** ]   [ **CSNK2A1** ]  mediated stimulation of rRT activity potently inhibited by glycyrrhetinic acid derivative.  Ref: 9881639 Biol Pharm Bull, 1998
- and iii this stimulation was selectively inhibited by potent CK II  [ **CSNK2B** ]   [ **CSNK2A1** ]  inhibitors, such as neocarzinostatin chromophore NCS chrom and three polyphenol containing anti oxidant compounds quercetin, epigallocatechin gallate EGCG and 8 chloro, tetrahydroxyisoflavone 8C, THI.  Ref: 10549869 Biol Pharm Bull, 1999
- Previous studies indicated that inositol hexasulphate inhibited the activity of CK I and or CK II  [ **CSNK2B** ]   [ **CSNK2A1** ]  in mouse tooth germs Torres Quintana et al.  Ref: 11077997 J Dent Res, 2000
- One unit of CK II  [ **CSNK2B** ]   [ **CSNK2A1** ]  activity is defined as the amount catalysing the transfer of 1 pmol 32P into the substrate within 1 minute at 37 degrees C.  Ref: 12017291 Anticancer Res< MedlineTA>Anticancer Res, 2000
- The reading frame encodes a polypeptide consisting of 526 amino acid residues, containing a number of DNA binding motifs and phosphorylation sites for PKC, CK II  [ **CSNK2B** ]   [ **CSNK2A1** ] , and p34cdc2.  Ref: 12736779 J Mol Med< MedlineTA>J Mol Med, 2000
- In contrast, CK II  [ **CSNK2B** ]   [ **CSNK2A1** ]  activity was significantly stimulated by lower doses 0.3 3 microl of GL, but was inhibited at high doses above 30 microM.  Ref: 10706406 Biol Pharm Bull, 2000
- Incubation of VT with purified Rhodnius CK II  [ **CSNK2B** ]   [ **CSNK2A1** ]  and 32 P ATP led to the incorporation of 2 mols of phosphate mol VT.  Ref: 12110292 Insect Biochem Mol Biol, 2002
- BACKGROUND Casein kinase II CK II  [ **CSNK2B** ]   [ **CSNK2A1** ]  is expressed at a higher level in lung tumours when compared to the corresponding non neoplastic lung tissue.  Ref: 12017291 Anticancer Res< MedlineTA>Anticancer Res, 2002
- These results indicate that phosphorylation at the CK II  [ **CSNK2B** ]   [ **CSNK2A1** ]  site is one of the post translational mechanisms through which p53 is activated in response to UV radiation.  Ref: 9501176 Proc Natl Acad Sci U S A, 1998
- AG114 174 mumol.L 1 and AG1109 126 mumol.L 1 had inhibitory effects on the activity of CK II  [ **CSNK2B** ]   [ **CSNK2A1** ]  p <.  Ref: 9812723 Zhongguo Yao Li Xue Bao, 1996
- Residual phosphorylation of the endogenous AGT by the PKs present in cell homogenates and phosphorylation of the recombinant AGT by purified serine threonine kinases, PKA, PKC, and CK II  [ **CSNK2B** ]   [ **CSNK2A1** ]  reduced AGT activity by 30 65%.  Ref: 10667577 Cancer Res, 2000
- CK II  [ **CSNK2B** ]   [ **CSNK2A1** ]  activity increases on day 1 after fertilization and reaches a plateau on day 7 and its activity remains elevated until eclosion.  Ref: 10319435 Insect Biochem Mol Biol, 1999
- Moreover the enzyme was found to be highly resistant to heparin, a potent inhibitor of casein kinase II CK II  [ **CSNK2B** ]   [ **CSNK2A1** ]  and also resistant to CK I 7, a synthetic inhibitor of CK I, but very sensitive to a bioflavonoid quercetin.  Ref: 9300039 Virology, 1997
- the nonsegmented negative strand RNA viruses, all essentially dependent on host cell kinases, e.g., protein kinase CK II  [ **CSNK2B** ]   [ **CSNK2A1** ]  casein kinase II, for which good inhibitors, such as halogenated benzimidazoles and benzotriazoles, are known.  Ref: 10454209 Pharmacol Ther< MedlineTA>Pharmacol Ther, 1997
- These enriched CK II  [ **CSNK2B** ]   [ **CSNK2A1** ]  fractions were assayed against different effectors, such as cAMP, H 8, H 89, calphostin C, sphingosine, polylysine and heparin.  Ref: 10319435 Insect Biochem Mol Biol, 1999
- RESULTS AG213 inhibited the activity of CK II  [ **CSNK2B** ]   [ **CSNK2A1** ]  with IC50 44.7 mumol.L 1 41.5 47.9 mumol.L 1, and AG1394 144 mumol.L 1 strongly inhibited the activity of CK II  [ **CSNK2B** ]   [ **CSNK2A1** ]  with an inhibitory ratio of 89%.  Ref: 9812723 Zhongguo Yao Li Xue Bao, 1996
- Thus, our data demonstrate that cellular CK II  [ **CSNK2B** ]   [ **CSNK2A1** ]  phosphorylates rabies virus N.  Ref: 12711319 Biochem Biophys Res Commun, 2003
- LFs to a glycyrrhizin GL affinity column and their phosphorylation by casein kinase II CK II  [ **CSNK2B** ]   [ **CSNK2A1** ]  in vitro were biochemically investigated.  Ref: 11041245 Biol Pharm Bull, 2000
- ii the PR activity of rPR is stimulated approximately 2.9 fold after its full phosphorylation by recombinant human CK II  [ **CSNK2B** ]   [ **CSNK2A1** ]  rhCK II in a manner similar to that observed for recombinant HIV 1 reverse transcriptase rRT.  Ref: 10964683 Biochem Biophys Res Commun, 2000
- However, the mass spectra of the tail fragments of the phosphorylated NF L indicated that CK II  [ **CSNK2B** ]   [ **CSNK2A1** ]  was the kinase mediating Ser 473 phosphorylation in vitro as opposed to CK I, because CK I phosphorylated another site as well as Ser 473 in vitro.  Ref: 10428477 FEBS Lett, 1999
- Enhanced casein kinase II CK II  [ **CSNK2B** ]   [ **CSNK2A1** ]  activity in human lung tumours.  Ref: 12017291 Anticancer Res< MedlineTA>Anticancer Res, 1999
- The physiological correlation between glycyrrhizin GL and high mobility group proteins I and 2 HMG1 2 and the inhibitory effect of GL on their phosphorylation by three protein kinases CK I, CK II  [ **CSNK2B** ]   [ **CSNK2A1** ]  and PKC were investigated biochemically in vitro.  Ref: 11510483 Biol Pharm Bull, 2001
- and ii CK II  [ **CSNK2B** ]   [ **CSNK2A1** ]  mediates stimulation of its PLA2 activity in vitro.  Ref: 11558558 Biol Pharm Bull, 2001
- All three activities correspond to known IkappaB kinases the mitogen activated 90 kDa ribosomal S6 kinase p90rsk1, the IkappaB kinase 1 2 complex IKK1 2 and casein kinase II CK II  [ **CSNK2B** ]   [ **CSNK2A1** ] .  Ref: 9914500 Eur J Biochem, 1999
- These results suggest that i CK II  [ **CSNK2B** ]   [ **CSNK2A1** ]  may be responsible for activation of RT in HIV 1 infected cells.  Ref: 10549869 Biol Pharm Bull, 1999
- It was found that recombinant Rev rRev and the synthetic oligo fragments corresponding to the amino acid sequences of its Arg rich cluster stimulate CK II  [ **CSNK2B** ]   [ **CSNK2A1** ]  activity in a dose dependent manner.  Ref: 9654140 FEBS Lett, 1998
- The stimulatory effects of several DNA binding basic proteins histone and protamine and HIV 1 Rev with arginine Arg rich clusters on the activity of casein kinase II CK II  [ **CSNK2B** ]   [ **CSNK2A1** ]  were investigated in vitro.  Ref: 9654140 FEBS Lett, 1998
- These results suggest that the GL induced inhibition of the DNA binding affinity and the CK II  [ **CSNK2B** ]   [ **CSNK2A1** ]  mediated phosphorylation of hLF may be closely correlated with the anti inflammatory effect of GL in the human body.  Ref: 11041245 Biol Pharm Bull, 2000
- These results suggest that i GL affinity column chromatography is useful for the selective purification of 60S acidic ribosomal P proteins as a heterocomplex associated with CK II  [ **CSNK2B** ]   [ **CSNK2A1** ]  from various cell sources.  Ref: 10443459 Biol Pharm Bull, 1999
- Determination of the N terminal amino acid sequences and immunological tests confirmed that these three CK II  [ **CSNK2B** ]   [ **CSNK2A1** ]  phosphate acceptors belong to the family of 60S acidic ribosomal proteins P0, P1 and P2.  Ref: 10706406 Biol Pharm Bull, 2000
- Casein kinase II CK II  [ **CSNK2B** ]   [ **CSNK2A1** ]  plays an important role in serine threonine dependent protein phosphorylation.  Ref: 9178953 Neurochem Res, 1997
- Using this antibody, we showed that p53 is phosphorylated at the CK II  [ **CSNK2B** ]   [ **CSNK2A1** ]  site upon UV treatment of early passage rat embryo fibroblasts and RKO cells.  Ref: 9501176 Proc Natl Acad Sci U S A, 1998
- In this study we show that Vitellin VT phosphorylation in chorionated oocytes of Rhodnius prolixus is completely inhibited by heparin 10 microg ml, a classical casein kinase II CK II  [ **CSNK2B** ]   [ **CSNK2A1** ]  inhibitor.  Ref: 12110292 Insect Biochem Mol Biol, 2002
- 0.25 microM and casein kinase II CK II  [ **CSNK2B** ]   [ **CSNK2A1** ] , ID 50 = approx.  Ref: 11226880 J Biochem Tokyo , 2001
- Furthermore, N phosphorylation in the virus infected cells can be inhibited by a CK II  [ **CSNK2B** ]   [ **CSNK2A1** ]  specific inhibitor, dichloro beta D ribofuranosyl benzimidazole.  Ref: 12711319 Biochem Biophys Res Commun, 2003
- CK II  [ **CSNK2B** ]   [ **CSNK2A1** ]  activity was assayed in each fraction and the enzyme obtained from the 3 day old eggs was shown to be three times more active than that obtained from oocytes, although the amount of enzyme present in the fractions was the same.  Ref: 10319435 Insect Biochem Mol Biol, 1999
- These data show that an insect yolk protein contain phosphorylation sites for a cyclic nucleotide independent protein kinase such as CK II  [ **CSNK2B** ]   [ **CSNK2A1** ] .  Ref: 12110292 Insect Biochem Mol Biol, 2002
- These results indicate that the HSV 2 UL13 PK had unique catalytic properties different from those of cellular CK I, CK II  [ **CSNK2B** ]   [ **CSNK2A1** ] , and the viral PK encoded by the US3 gene.  Ref: 9300039 Virology, 1997
- The physiological significance of casein kinase II CK II  [ **CSNK2B** ]   [ **CSNK2A1** ]  on the protease PR activity of recombinant HIV 1 PR rPR was biochemically investigated in vitro.  Ref: 10964683 Biochem Biophys Res Commun, 2000
- This result may support the suggestion that CK II  [ **CSNK2B** ]   [ **CSNK2A1** ]  is the kinase responsible for Ser 473 phosphorylation.  Ref: 10428477 FEBS Lett, 1999
- We found that i the purified rPR p11 functions as a phosphate acceptor of CK II  [ **CSNK2B** ]   [ **CSNK2A1** ] .  Ref: 10964683 Biochem Biophys Res Commun, 2000
- In addition, the phosphorylation of the recombinant N in vitro can be blocked by a CK II  [ **CSNK2B** ]   [ **CSNK2A1** ]  inhibitor, heparin.  Ref: 12711319 Biochem Biophys Res Commun, 2003
- When CK II  [ **CSNK2B** ]   [ **CSNK2A1** ]  activity was assayed in non fertilized eggs, no activation of the enzyme was observed when compared to fertilized eggs.  Ref: 10319435 Insect Biochem Mol Biol, 1999
- and iv oGA as well as NCS chromophore effectively prevented the CK II  [ **CSNK2B** ]   [ **CSNK2A1** ]  mediated stimulation of RDDP activity.  Ref: 9881639 Biol Pharm Bull, 1998
- AG555 136 mumol.L 1 had little effect on CK II  [ **CSNK2B** ]   [ **CSNK2A1** ]  activity.  Ref: 9812723 Zhongguo Yao Li Xue Bao, 1996
- Quercetin was found to be the most effective compound on CK II  [ **CSNK2B** ]   [ **CSNK2A1** ]  activity since its ID50 was approx.  Ref: 10443459 Biol Pharm Bull, 1999
- Biochemical characterization of 60S acidic ribosomal P proteins associated with CK II  [ **CSNK2B** ]   [ **CSNK2A1** ]  from bamboo shoots and potent inhibitors of their phosphorylation in vitro.  Ref: 10443459 Biol Pharm Bull, 1999
- The recombinant N was phosphorylated by BHK cellular extracts and by purified CK II  [ **CSNK2B** ]   [ **CSNK2A1** ] .  Ref: 12711319 Biochem Biophys Res Commun, 2003
- We have obtained a 3000 fold VT free enriched preparation of CK II  [ **CSNK2B** ]   [ **CSNK2A1** ] .  Ref: 12110292 Insect Biochem Mol Biol, 2002
- Characterization of a CK II  [ **CSNK2B** ]   [ **CSNK2A1** ]  protein kinase from etiolated oat seedlings.  Ref: 12243354 Mol Cells, 2002
- These properties identify the major protein kinase activity as a type II casein kinase CK II  [ **CSNK2B** ]   [ **CSNK2A1** ] .  Ref: 11446384 Insect Biochem Mol Biol, 1993
- Inhibition of CK II  [ **CSNK2B** ]   [ **CSNK2A1** ]  mediated phosphorylation of functional cellular proteins, including calmodulin CaM, by actin was significantly stimulated after its full phosphorylation by the purified 37 kDa actin kinase or rCK I in vitro.  Ref: 10564775 Biochim Biophys Acta, 1999
- MATERIALS AND METHODS The aim of the present study was to evaluate the activity of CK II  [ **CSNK2B** ]   [ **CSNK2A1** ]  both in neoplastic and non neoplastic human lung tissue by using a synthetic peptide with Arg Arg Arg Asp Asp Asp Ser Asp Asp Asp RRRDDDSDDD sequence.  Ref: 12017291 Anticancer Res< MedlineTA>Anticancer Res, 1999
- Therefore, both p90rsk1 and CK II  [ **CSNK2B** ]   [ **CSNK2A1** ]  have the same preference for phosphorylating only one of the two serines which are relevant for inducible degradation.  Ref: 9914500 Eur J Biochem, 1999
- and 2 specific phosphorylation of actin by the actin kinase may be involved in the suppression mechanism of CK II  [ **CSNK2B** ]   [ **CSNK2A1** ]  mediated signal transduction at the cellular level.  Ref: 10564775 Biochim Biophys Acta, 1999
- The data presented here suggest that HIV 1 Rev acts as an effective potent activator of CK II  [ **CSNK2B** ]   [ **CSNK2A1** ] , which may be a cellular mediator promoting HIV 1 replication in virus infected cells.  Ref: 9654140 FEBS Lett, 1998
- Casein kinase II CK II  [ **CSNK2B** ]   [ **CSNK2A1** ]  incorporated 4 and 2 mol of phosphate mol of MIIB alpha F47 and MIIB beta F47 peptides, respectively, and this caused strong inhibition of assembly for MIIB alpha F47 but only slight inhibition for MIIB beta F47.  Ref: 9485326 Biochemistry, 1998
- AIM To investigate the effects of tyrphostins, AG213, AG1394, AG114, AG1109, AG555 on the activity of casein kinase CK II  [ **CSNK2B** ]   [ **CSNK2A1** ] .  Ref: 9812723 Zhongguo Yao Li Xue Bao, 1996
- We compared the properties of the intrinsic A170 kinases with those of mitogen activated protein kinase ERK 2, protein kinase A PKA, casein kinase II CK II  [ **CSNK2B** ]   [ **CSNK2A1** ] , and protein kinase C, since their catalytic subunits have molecular masses similar to A170 kinases.  Ref: 9405250 Biochem Biophys Res Commun, 1997
- Protein kinase casein kinase II CK II  [ **CSNK2B** ]   [ **CSNK2A1** ]  activity was assayed during Rhodnius prolixus embryogenesis.  Ref: 10319435 Insect Biochem Mol Biol, 1999
- Using the multifunctional protein kinase CK II  [ **CSNK2B** ]   [ **CSNK2A1** ] , we have shown that incorporation of phosphate into P1A protein can exert its effect on the hetero oligomerization process, namely by preventing the formation of the hetero oligomer P1A P P2B.  Ref: 11118639 Biochim Biophys Acta, 2000
- CONCLUSION Some tyrphostins are potent inhibitors of CK II  [ **CSNK2B** ]   [ **CSNK2A1** ] .  Ref: 9812723 Zhongguo Yao Li Xue Bao, 1996
- These results suggest that the beta subunit of CF1 is a substrate protein of CK II  [ **CSNK2B** ]   [ **CSNK2A1** ]  in the chloroplast.  Ref: 9784844 Biochem Mol Biol Int, 1998
- Both AG372 121 mumol.L 1 and AG1112 150 mumol.L 1 displayed inhibitory effects on the activity of CK II  [ **CSNK2B** ]   [ **CSNK2A1** ] .  Ref: 10072895 Zhongguo Yao Li Xue Bao, 1997
- Phosphorylation by protein kinase C PK C or casein kinase II CK II  [ **CSNK2B** ]   [ **CSNK2A1** ]  within or near the nonhelical tail end domain inhibits assembly of MIIB alpha F47 Murakami, N.  Ref: 10985790 Biochemistry, 2000
- The 40 and 44 kDa kinases present in the macrophage extract were similar to alpha and alpha subunits of CK II  [ **CSNK2B** ]   [ **CSNK2A1** ]  in respect to substrate specificity, pharmacological properties, immuno reactivities, and ubiquitous expression in tissues.  Ref: 9405250 Biochem Biophys Res Commun, 1997
- Two dihydroflavonol rhamnosides 1 and 2 isolated from the bark of Hymenaea parvifolia and two pentacyclic triterpenoids 3 and 6 obtained from the leaves of Wulffia baccata have been found to exhibit inhibitory effects of casein kinase II CK II  [ **CSNK2B** ]   [ **CSNK2A1** ]  dose dependently, suggesting that at higher doses more than 10 microM, these four compounds may act as potent CK II  [ **CSNK2B** ]   [ **CSNK2A1** ]  suppressors of the CK II  [ **CSNK2B** ]   [ **CSNK2A1** ]  mediated activation of 60S acidic ribosomal P proteins in vitro.  Ref: 10465536 Bioorg Med Chem Lett, 1999
- ERK 2, CK II  [ **CSNK2B** ]   [ **CSNK2A1** ] , and PKA phosphorylated recombinant A170 as a substrate.  Ref: 9405250 Biochem Biophys Res Commun, 1997
- CK II  [ **CSNK2B** ]   [ **CSNK2A1** ]  was associated with p35 since the GL affinity fraction was coprecipitated with an anti serum against Drosophila CK IIbeta.  Ref: 10443459 Biol Pharm Bull, 1999
- The respective serines are located in conserved recognition sites for luminal protein kinases protein kinase CK II  [ **CSNK2B** ]   [ **CSNK2A1** ]  and Golgi casein kinase.  Ref: 12033762 Virology, 2002
- The monoclonal antibodies CK II  [ **CSNK2B** ]   [ **CSNK2A1** ]  and A45 B B3 were used with the immuno cytochemical standard method for detecting IDT BM.  Ref: 12678519 Int J Surg Investig, 2000
- Cytosolic CK II  [ **CSNK2B** ]   [ **CSNK2A1** ]  activity was also unaffected.  Ref: 9178953 Neurochem Res, 1997
- 50 nM was found to be an effective CK II  [ **CSNK2B** ]   [ **CSNK2A1** ]  inhibitor.  Ref: 10706406 Biol Pharm Bull, 2000
- Additionally, we show here that human PHI is phosphorylated at serine 185 by casein kinase II CK II  [ **CSNK2B** ]   [ **CSNK2A1** ]  and we provide experimental evidence suggesting that this phosphorylation is associated with secretion, thus providing insights for elucidating the intracellular signal transmission of cell response to stimulation by AMF NLK MF.  Ref: 11004567 Biochim Biophys Acta, 2000
